# Predictors of non-transplantation of adult donor organs - an observational study using routine data from Eurotransplant

**DOI:** 10.1186/s12913-014-0584-3

**Published:** 2014-11-25

**Authors:** Karl Philipp Drewitz, Martin Loss, Julika Loss, Christian Joachim Apfelbacher

**Affiliations:** Medical Sociology, Institute of Epidemiology and Preventive Medicine, University of Regensburg, Dr.-Gessler-Str. 17, 93051 Regensburg, Germany; Department of Surgery, University Hospital Regensburg, Franz-Josef-Strauss-Allee 11, 93051 Regensburg, Germany

**Keywords:** Pancreas transplantation, Donor factors, Donor organ allocation

## Abstract

**Background:**

The majority of pancreases, offered in allocation, are not transplanted. This pancreas under-utilisation is a phenomenon observed in all transplant systems in North-America and Europe. It was the aim of this study to analyse factors predictive of pancreas non-transplantation in Germany.

**Methods:**

Routine Eurotransplant data of 3,666 deceased German donors (from 2002–2011) were used for multivariate modelling. Socio-demographic and medical factors were considered as independent variables in logistic regression models with non-transplantation as dependent variable.

**Results:**

Male gender, advanced age, overweight/obesity, long ICU stay, a history of smoking, non-traumatic brain death, elevated levels of sodium, serum glucose, lipase/amylase and the liver not being considered for procurement were significant independent predictors of non-transplantation.

**Conclusion:**

In line with previous research, advanced age, high BMI, long ICU stay and the liver not being considered for procurement were the strongest predictors of pancreas non-transplantation in Germany. About three quarters of the variance remained unexplained, suggesting that factors not assessed or unknown may play a decisive role.

## Background

Pancreas or islet transplantation is currently the only means to restore normal glucose metabolism and substantially improves quality of life in patients with type 1 diabetes [1,2]. In patients with diabetes mellitus and end-stage renal disease, simultaneous pancreas-kidney transplantation is associated with decreased mortality compared with patients on the waiting list or deceased kidney-only transplant recipients [3-6]. Not all patients eligible for pancreas transplantation benefit from this therapeutic option which is commonly linked to a shortage of donor organs. Less emphasis has been put on the fact that not all donor pancreases offered for allocation are appropriately used. Pancreas underutilization has been reported for the United States [7] and the Eurotransplant (ET) region [8].

Using routine data from ET we have previously analysed physician-reported refusal reasons to better understand why offered pancreases are refused in the allocation process [9]. A multitude of refusal reasons has been reported which were grouped in four domains: donor-related criteria, recipient-related criteria, logistic criteria and technical criteria. Overall, the significance of various refusal reasons was not judged in a consistent manner and often, refusal reasons seemed to lack plausibility in the absence of evidence supporting the refusal. Some donor characteristics were quite unambiguous reasons for discarding a pancreas. For example, among pancreases for which ‘diabetes mellitus in the donor’, ‘malignancy in the donor’ or ‘CIT’ were named at least once, the proportion of finally transplanted organs was below 10% (4, 7, and 8%, respectively). Other criteria were obviously less distinct: for instance, organs refused at least once for ‘trauma in donor’, ‘donor age’, or ‘resuscitation’ were later transplanted in 48%, 32%, and 28% of cases, respectively.

In light of these findings, it is of interest to understand which objective donor-related factors are predictive of non-transplantation.

Therefore, the aim of this study was to investigate the relative importance of relevant socio-demographic and medical donor characteristics in determining non-transplantation using a large sample from the ET-area for 2002–2011.

## Methods

### Data source

The analysis is based on the database in which ET routinely collects medical and socio-demographic information on deceased potential organ donors during the allocation procedure. A detailed description of the sampling frame is given elsewhere [9].

Briefly, the study population is composed of all German pancreas donors reported between Jan 1^st^ 2002 and Dec 31^st^ 2011, whose organs were offered for allocation. According to previous studies we included all donors older than 10 years as they could be classified as 'adult' donors [10-13]. We excluded those donors where pancreatic islet cell transplantation was intended or performed in the end. We also excluded all donors with a positive test for Human Immunodeficiency Virus (HIV), since an HIV infection represents a general contraindication for pancreas donation. 3,666 donors aged 11–69 years were included.

### Predictive variables

The following variables pertaining to the social and medical history of the donors as well as technical and logistic information were included for analysis:

- donor gender [male/female]
- body mass index (BMI) [kg/m^2^], based on weight/height^2^ and formed categories for <19, 20–24, 25–29 and ≥30
- age, at the time of brain death, categorized in 7 age groups for 11–19, 20–29, 30–39, 40–44, 45–49, 50–54 and ≥55 years
- duration of the ICU stay (based on date of registration minus date of admission to the ICU), categorized as 0–2, 3–7, 8–11, 12–15 and ≥16 days; values >32 days were considered implausible and therefore classified as missing
- serum level of amylase [U/l], categorized as low (<130), elevated (130–389) and highly elevated (≥390)
- serum level of lipase [U/l], categorized as low (<160), elevated (160–479) and highly elevated (≥480)
- serum level of lipase/amylase [U/l], categorized as low, elevated and highly elevated where categories for lipase were used if available and supplemented by categories for amylase if lipase values were not available
- serum level of sodium [mmol/l], categorized as <120, 120–134, 135–145, 146–159 and ≥160
- serum level of blood glucose [mg/dl], categorized as <100, 100–130, 131–180, 181–209 and ≥210; values lower than 50 and higher than 300 were considered implausible and therefore classified as missing
- glycated hemoglobin (HbA1c) in % of total haemoglobin; values >40 were considered implausible and therefore classified as missing
- cause of death [traumatic/non-traumatic], based on the documented ICD-10 diagnosis
- catecholamine administration [yes/no], based on the use of dopamine/γ or adrenalin/γ
- virology statement [yes/no], if any of CMV, HBV or HCV was positive
- history of smoking [yes/no] based on medical history by proxy
- donor region [region 1–7 in Germany]
- year of registration [2002–2011]
- information on the liver, if it was considered for allocation as an indicator for the possibility to procure abdominal organs [yes/no] [11,14,15].

The factors were selected based on a review of the literature and previous findings on differences between donors of transplanted and non-transplanted pancreases [9,16].

### Outcome variable

Outcome was defined as non-transplantation of the pancreas.

### Missing values

There were no missing values for donor region, donor gender, donor weight/height, date of birth, date/time of brain death. In 203 cases the date/time of admission to ICU was not available, or the computed length of ICU stay was negative or beyond 31 days. These values were classified as ‘missing’.

### Data management and statistical analysis

Using Microsoft Excel, a database was compiled which included donors’ socio-demographic and medical information. Statistical analyses were undertaken using SAS version 9.0 WIN (Cary NC, USA). Median and range were computed for continuous variables due to evidence for non-normality of the considered variables. Counts and per cent were computed for categorical variables. Donor factors were compared between donors whose pancreas was discarded and donors whose pancreas was transplanted, using Pearson’s Chi-square-test. Since donor age, length of ICU stay and the considered lab values were not normally distributed, differences in these continuous variables were tested using the non-parametric Mann–Whitney-U-Test. A p-value below 0.05 was considered statistically significant.

Univariate logistic regression was used to explore the relationship between each of the explanatory variables described above and pancreas non-transplantation. Multivariate logistic regression was then used to explore the independent effects of the explanatory variables. We obtained crude and adjusted odds ratios (ORs) and 95% confidence intervals (CIs). For our full model we included all continuous and categorical items listed above. Stratified analyses were performed for donor gender. In order to arrive at parsimonious models we performed backward selection (p-value for exclusion: 0.1). All analyses are adjusted for donor region and year of donation.

### Ethical approval

As no personal data for identification of (potential) pancreas donors were visible, no authorized approval was needed as per the ethics committee of the University of Regensburg (reference number 11-160-0192).

## Results

Of the 3,666 evaluated donor organs 2,232 (60.9%) were discarded. Of the utilized pancreases, 1,268 (88.4%) were transplanted together with a kidney (simultaneous pancreas kidney transplantation, SPK), 135 (9.4%) as pancreas transplantation alone (PTA) or pancreas after kidney transplantation (PAK) and 31 (2.2%) in combination with heart, liver or intestine as “approved combined organ” (ACO) transplantation.

### Characteristics of the study population

Socio-demographic and medical characteristics of all donors are shown in Table 1. The median age of all donors was 40 years and there were more males in the sample than females. Median BMI was 24.1 kg/m^2^ and median duration of ICU stay was 3.2 days. For more than 40% of all donors a history of smoking was reported. Slightly more than half of all donors had a positive virology statement (Hepatitis B or C or Human Cytomegalovirus) and around 39.6% had a traumatic cause of brain death. Catecholamine was administered in around 78.4%. The pancreas was procured in 54.8%.

**Table 1:** **Donor characteristics of all donors**

	**All donors (n = 3,666)**	**N**
Age (mean, years)	36.97	3666
Age (median, years)	40.08	
Age (range, years)	11-69	
Donors 11 < years < 20 (%)	11.35	
Donors >45 years (%)	30.58	
Positive virology statement (%)	53.80	3656
Male (%)	56.11	3666
Traumatic Cause of brain death (%)	39.61	3663
BMI (mean, kg/m^2^)	24.11	
BMI (median, kg/m^2^)	24.07	
BMI (range, kg/m^2^)	11-42	
Liver NOT reported (%)	0.74	3666
P-PASS (median)	17.58	3408
P-PASS (range)	9-25	
ICU stay (mean, days)	4.87	3463
ICU stay (median, days)	3.19	
ICU stay (range, days)	0-31	
ICU stay >7 days (%)	19.90	
History of smoking	43.25	3082
Catecholamine administration (%)	78.40	3666
Pancreas procured (%)	54.75	3666
Sodium (mean, mmol/L)	147.08	3663
Sodium (median, mmol/L)	147.00	
Sodium (range, mmol/L)	6-193	
Lipase (mean, U/l)	770.34	3020
Lipase (median, U/l)	35.00	
Lipase (range, U/l)	0-45383	
Amylase (mean, U/l)	530.20	2899
Amylase (median, U/l)	86.00	
Amylase (range, U/l)	0-47119	
Serum glucose (mean, mg/dl)	140.21	3243
Serum glucose (median, mgl/dl)	131.00	
Serum glucose (range, mg/dl)	50-300	
HbA1c (mean,%)	5.46	577
HbA1c (median,%)	5.40	
HbA1c (range,%)	0.01-37.00	

BMI: Body Mass Index, ICU: Intensive Care Unit, U/L: Units Per Litre.

### Analysis of donor factors - univariate associations

The comparison of the above mentioned characteristics between those whose pancreas was discarded and those whose pancreas was transplanted is shown in Table 2. Median age, median BMI, median duration of ICU stay as well as median serum levels of sodium, glucose, lipase and amylase were significantly higher in those whose pancreas was discarded compared to those whose pancreas was transplanted. The prevalences of male gender, a history of smoking and a positive virology statement were significantly higher in those whose pancreas was discarded compared to those whose pancreas was transplanted. Donors whose organs were not transplanted deceased significantly more often from cerebro-vascular brain death than primarily traumatic brain death.

**Table 2:** **Comparison of donor characteristics**

	**Pancreases discarded (n = 2,232)**	**Pancreases transplanted (n = 1,434)**	**P**	**N**
Age (mean, years)	39.63	32.82		3666
Age (median, years)	42.61	33.68	<0.0001	
Age (range, years)	11-69	11-58		
Donors 11 < years < 20 (%)	7.17	17.85		
Donors >45 years (%)	38.22	18.69		
Positive virology statement (%)	55.66	50.91	0.005	3656
Male (%)	57.53	53.91	0.0311	3666
Traumatic Cause of brain death (%)	34.59	47.42	<0.0001	3663
BMI (mean, kg/m^2^)	24.58	23.36		
BMI (median, kg/m^2^)	24.49	23.24	<0.0001	
BMI (range, kg/m^2^)	12-42	11-35		
Liver NOT reported (%)	1.03	0.28	0.0094	3666
P-PASS (median)	19.00	17.00	<0.0001	3408
P-PASS (range)	10-25	9-24		
ICU stay (mean, days)	5.62	3.69		3463
ICU stay (median, days)	3.94	2.58	<0.0001	
ICU stay (range, days)	0-31	0-31		
ICU stay >7 days (%)	25.84	10.64	<0.0001	
History of smoking	47.62	36.71	<0.0001	3082
Catecholamine administration (%)	78.76	77.82	0.5002	3666
Pancreas procured (%)	25.67	100	<0.0001	3666
Sodium (mean, mmol/L)	147.57	146.30		3663
Sodium (median, mmol/L)	147.00	146.00	<0.0001	
Sodium (range, mmol/L)	6-193	60-189		
Lipase (mean, U/l)	733.30	828.34		3020
Lipase (median, U/l)	40.00	29.00	<0.0001	
Lipase (range, U/l)	0-45383	0-42767		
Amylase (mean, U/l)	585.49	444.03		2899
Amylase (median, U/l)	92.00	82.00	0.0008	
Amylase (range, U/l)	0-44287	6-47119		
Serum Glucose (mean, mg/dl)	142.11	137.25		3243
Serum Glucose (median, mg/dl)	133.33	127.00	0.0078	
Serum Glucose (range, mg/dl)	50.00-300.00	54.05-294.00		
HbA1c (mean, %)	5.52	5.35		577
HbA1c (median, %)	5.40	5.40	0.1453	
HbA1c (range, %)	0.01-37.00	2.50-6.70		

### Analysis of donor factors – multivariate associations

In multivariate analysis (see Table 3), male gender, history of smoking, a primarily non-traumatic cause of brain death (e.g. stroke), a longer ICU stay and liver not being considered for donation or procurement showed significant positive associations with non-transplantation. The chance of a pancreas not being transplanted increased for each age group from 30–39 years up to the group 55 years and older and also increased with weight for height (BMI) category. High levels of sodium and blood glucose were significantly associated with non-transplantation as well as elevated or highly elevated levels of either lipase or amylase. Catecholamine administration was not significantly related to non-transplantation. 26% of the variance was explained by the variables considered.

**Table 3:** **Adjusted full model**

**Variables**	**Description**	**Full model**			
		***−2LL: 2774, adj. r*** ^***2***^ ***: 0.26***			**N**
					**2441**
		**OR**	**95%-CI**		
Gender	male vs female	1.57	1.29	1.91	
Age	11-19 vs 20–29 yrs	1.06	0.75	1.49	
	30-39 vs 20–29 yrs	1.80	1.35	2.40	
	40-44 vs 20–29 yrs	2.64	1.96	3.56	
	45-49 vs 20–29 yrs	3.49	2.59	4.71	
	50-54 vs 20–29 yrs	4.53	2.84	7.23	
	55 and older vs 20–29 yrs	91.61	12.12	692.24	
BMI	0-19 vs 20-24	1.12	0.75	1.66	
	25-29 vs 20-24	1.98	1.60	2.44	
	30 and more vs 20-24	3.13	1.32	7.39	
ICU stay	3-7 vs 0–2 days	1.48	1.21	1.82	
	8-11 vs 0–2 days	2.69	1.96	3.69	
	12-15 vs 0–2 days	4.96	2.94	8.37	
	16 and longer vs 0–2 days	4.85	2.41	9.78	
History of smoking	Smoking Yes vs No	1.32	1.10	1.60	
Cause of brain death	traumatic brain death No vs Yes	1.28	1.04	1.58	
Sodium	Sodium <120 vs 135-145	2.17	0.26	23.97	
	Sodium 120–134 vs 135-145	0.97	0.66	1.42	
	Sodium 146–159 vs 135-145	1.14	0.94	1.39	
	Sodium 160 < vs 135-145	1.75	1.18	2.61	
Serum blood glucose	Glucose <100 vs 100-130	1.23	0.93	1.62	
	Glucose 131–180 vs 100-130	1.18	0.95	1.47	
	Glucose 181–209 vs 100-130	0.90	0.61	1.31	
	Glucose 210 < vs 100-130	1.76	1.21	2.58	
Lipase/Amylase	if at least one value was given**				
	elevated vs low	1.39	1.03	1.87	
	highly elevated vs low	2.06	1.27	3.33	
Virology	min. 1 positive virol. Statement	removed/backward-selection			
Catecholamine	administration Yes vs No	removed/backward-selection			
Status of liver	Liver not reported vs liver reported	4.95	1.01	24.13	

**Amylase categorized as low (<130), elevated (130–389) and highly elevated (≥390); Lipase categorized as low (<160), elevated (160–479) and highly elevated (≥480); the categories were chosen if either one of the enzymes scored elevated/highly elevated.

### Subgroup analyses

In gender-stratified analyses (see Table 4), both overweight and obesity as well as prolonged ICU stay showed stronger associations with non-transplantation in female donors than in male donors. No substantial differences in effect estimates were observed for age group and lipase/amylase although the significant associations with lipase/amylase were only found for highly elevated values. History of smoking was only significantly related to non-transplantation in male donors. The remainder of the considered variables could not be compared due to variables being removed in either one or both models: cause of brain death and serum sodium level were removed for the model encompassing females and serum blood glucose for the model including males after backward selection. Virology statement, catecholamine administration and the status of the liver were removed in both groups. 24% of the variance was explained in the model for females while 28% of the variance was explained in the model for male donors.

**Table 4:** **Adjusted full models according to gender**

**Variables**	**Description**	**Adjusted/stratified by gender**							
		**Female donors**				**Male donors**			
		**−2LL: 1475, adj. r** ^**2**^ **: 0.24**			**N**	**−2LL: 1493, adj. r** ^**2**^ **: 0.28**			**N**
					**1081**				**1360**
		**OR**	**95%-CI**			**OR**	**95%-CI**		
Age	11-19 vs 20–29 yrs	1.40	0.76	2.58		0.94	0.62	1.42	
	30-39 vs 20–29 yrs	1.46	0.90	2.36		2.16	1.50	3.11	
	40-44 vs 20–29 yrs	2.00	1.24	3.24		3.60	2.41	5.38	
	45-49 vs 20–29 yrs	2.64	1.66	4.21		4.67	3.07	7.09	
	50-54 vs 20–29 yrs	5.06	2.55	10.06		3.90	2.02	7.52	
	55 and older vs 20–29 yrs	54.12	6.66	439.73		N/A			
BMI	0-19 vs 20-24	1.03	0.63	1.68		1.40	0.71	2.76	
	25-29 vs 20-24	2.52	1.81	3.52		1.66	1.26	2.20	
	30 and more vs 20-24	4.00	1.30	12.30		2.56	0.65	10.02	
ICU stay	3-7 vs 0–2 days	1.65	1.21	2.24		1.40	1.07	1.84	
	8-11 vs 0–2 days	2.46	1.58	3.84		3.26	2.06	5.15	
	12-15 vs 0–2 days	6.34	3.25	12.38		4.18	1.77	9.87	
	16 and longer vs 0–2 days	6.57	2.54	16.98		3.77	1.29	11.07	
History of smoking	Smoking Yes vs No	1.31	0.98	1.74		1.37	1.06	1.77	
Cause of brain death	traumatic brain death No vs Yes	removed/backward-selection				1.42	1.09	1.85	
Sodium	Sodium <120 vs 135-145	removed/backward-selection				N/A			
	Sodium 120–134 vs 135-145					0.64	0.37	1.10	
	Sodium 146–159 vs 135-145					1.18	0.90	1.54	
	Sodium 160 < vs 135-145					1.71	1.02	2.86	
Serum blood glucose	Glucose <100 vs 100-130	1.43	0.93	2.18		removed/backward-selection			
	Glucose 131–180 vs 100-130	1.28	0.92	1.76					
	Glucose 181–209 vs 100-130	0.99	0.58	1.67					
	Glucose 210 < vs 100-130	2.71	1.54	4.74					
Lipase/Amylase	if at least one value was given**								
	elevated vs low	1.18	0.74	1.87		1.47	0.99	2.21	
	highly elevated vs low	2.20	1.07	4.50		2.06	1.09	3.91	
Virology	min. 1 positive virol. Statement	removed/backward-selection				removed/backward-selection			
Catecholamine	administration Yes vs No	removed/backward-selection				removed/backward-selection			
Status of liver	Liver not reported vs liver reported	removed/backward-selection				removed/backward-selection			

**Amylase categorized as low (<130), elevated (130–389) and highly elevated (≥390); Lipase categorized as low (<160), elevated (160–479) and highly elevated (≥480); the categories were chosen if either one of the enzymes scored elevated/highly elevated.

## Discussion

### Principal findings

In our analysis we identified several donor factors which were significantly associated with non-transplantation: increasing age, increasing ICU stay, overweight/obesity, liver not reported for procurement, history of smoking, male gender, a primarily non-traumatic cause of brain death and high levels of sodium, blood glucose, amylase and lipase. The focus of the study was the investigation of factors that were independently associated with non-transplantation and not the analysis of medical and non-medical reasons for organ refusal which have been previously reported [17].

The factors which showed the strongest association with non-transplantation of a donor pancreas were non-availability of the liver for procurement, prolonged ICU treatment, obesity and donor age >50 years. The high estimate linked to liver procurement might arise from the fact that the donors suffering from a severe abdominal trauma are generally not accepted for pancreas transplantation. In addition, liver not being reported for transplantation may indicate a poor general condition of the donor which may in turn account for the pancreas not being transplanted, too.

We also found effect modification by gender for BMI and duration of ICU stay. In both cases effect estimates were substantially stronger in female compared to male donors.

### Strengths and weaknesses of the study

Strengths of the study include the selection of a broad spectrum of variables as influencing factors, the use of multivariate regression modelling to assess independent effects and the large sample size. Differences over the years and between regions are accounted for as all multivariate models were adjusted for donor region and year of donation. To our knowledge, this is the largest study conducted so far comparing donors whose pancreas was discarded and donors whose pancreas was transplanted. Since Germany contributes ≈ 60% of all donated organs in ET [8,18], our findings are also relevant for the ET-area as a whole.

It must be acknowledged that we used routine data, so missing and implausible data could not be avoided in some cases which resulted in a slightly reduced sample. We did not look at long-term outcome of graft or patient survival because these data were not available when our analyses were carried out.

### Comparison to other studies

Other groups have previously performed similar comparisons, but only one study undertook analyses that are directly comparable to ours. In this study, data from consecutively reported pancreas donors for the period January 2002 to June 2005 were used to establish a prognostic score for the acceptance of a pancreas, the pre-procurement pancreas suitability score (P-PASS) [19]. 1,190 accepted pancreata were compared with 985 refused organs. In line with our findings, donors of pancreata that were not accepted were older, had fewer traumatic brain deaths, stayed on ICU for a longer period and had higher median values for sodium, amylase and lipase. In contrast to our study, Vinkers et al. did not find a difference in median BMI. They also reported that the gender distribution between groups was almost identical whereas we discovered the proportion of males to be significantly higher in those whose pancreas was discarded. As expected, median P-PASS was significantly higher in discarded pancreata compared to transplanted pancreata in our study but our values were higher compared to the P-PASS validation study (19.00/17.00 in our study vs 16.50/14.50).

Wullstein et al. compared accepted pancreas grafts and pancreas grafts refused for medical reasons, using all offered pancreata in the ET area between May 2002 and September 2003 [20]. As could be expected, significant differences were found for age, BMI, a history of smoking, serum glucose, amylase and lipase in the same direction as was shown by our analyses. However, their samples were older compared to our sample but a history of smoking was more frequently reported in our samples. In contrast to our findings, Wullstein et al. found the occurrence of a traumatic cause of brain death to be higher in those pancreas grafts that were refused for medical reasons. The comparison with the study by Wullstein et al. is hampered by the fact that we did not exclude organs that were refused for other than medical reasons.

Wiseman et al. examined Organ Procurement and Transplantation Network (OPTN) data from the US between 2005 and 2007 and compared 4,163 pancreas donors with 1,763 “potential pancreas donors” (PPD, defined by age 19–40 years, BMI < 30 kg/m^2^, successful liver donation and negative viral serology testing) who would typically result in organ acceptance but had not been accepted [15]. In accordance with our findings, a higher age, a higher proportion of head trauma and higher values for lipase and amylase were found in PPD compared to actual pancreas donors. Similarly to Vinkers et al. [19], and contrasting our findings, no significant difference was found for BMI. It is evident that a direct comparison with the study performed by Wiseman et al. is hampered by the fact that PPD is a subsample of all declined pancreata which we had fully considered in our analyses.

None of the papers considered for comparative purposes have included ICU stay in their analyses which in our analysis emerged as one of the factors that was strongly related to the outcome of non-transplantation. Hence we went further than previous studies. However, when considering the variance explained in our full model it becomes apparent that 74% of the variance remain to be explained. This suggests that yet unknown factors may play an influential role. Therefore, any attempt to establish clear criteria for organ quality based on comparisons between declined and accepted pancreata needs to be viewed with caution. We seem to lack knowledge on further factors that are important and powerful predictors. In this context, Wiseman et al. [15] refer to the severity of factors (such as smoking) which may play an important role but are not captured in the available data. He also points out that the available data in general do not well reflect the criteria that are in fact taken into account when accepting or rejecting an organ.

## Conclusion

Using routine ET data for German pancreas donors from 2002 to 2011, we present current data on factors determining pancreas non-transplantation and provide the most comprehensive analysis on this matter so far. We expand existing knowledge and have shown that in addition to advanced age and high BMI a long ICU stay and the liver not being considered for procurement were the strongest predictors of pancreas non-transplantation in Germany. The effects of BMI and ICU stay appear to be substantially stronger in females. The results are relevant for the ET region as a whole. About three quarters of the variance in multivariate analysis remained unexplained, suggesting that factors not assessed or unknown may play a decisive role.
